# Naltrexone is neuroprotective against traumatic brain injury in mu opioid receptor knockout mice

**DOI:** 10.1111/cns.13655

**Published:** 2021-05-21

**Authors:** Yu‐Syuan Wang, Tsai‐Wei Hung, Eun‐Kyung Bae, Kuo‐Jen Wu, Wei Hsieh, Seong‐Jin Yu

**Affiliations:** ^1^ Center for Neuropsychiatric Research National Health Research Institute Zhunan Taiwan

**Keywords:** inflammation, mu opioid receptor, naltrexone, traumatic brain injury

## Abstract

**Aims:**

Naltrexone is a mu opioid receptor (MOR) antagonist used to treat drug dependence in patients. Previous reports indicated that MOR antagonists reduced neurodegeneration and inflammation after brain injury. The purpose of this study was to evaluate the neuroprotective effect of naltrexone in cell culture and a mouse model of traumatic brain injury (TBI).

**Methods:**

The neuroprotective effect of naltrexone was examined in primary cortical neurons co‐cultured with BV2 microglia. Controlled cortical impact (CCI) was delivered to the left cerebral cortex of adult male MOR wild‐type (WT) and knockout (KO) mice. Naltrexone was given daily for 4 days, starting from day 2 after lesioning. Locomotor activity was evaluated on day 5 after the CCI. Brain tissues were collected for immunostaining, Western, and qPCR analysis.

**Results:**

Glutamate reduced MAP2 immunoreactivity (‐ir), while increased IBA1‐ir in neuron/BV2 co‐culture; both responses were antagonized by naltrexone. TBI significantly reduced locomotor activity and increased the expression of IBA1, iNOS, and CD4 in the lesioned cortex. Naltrexone significantly and equally antagonized the motor deficits and expression of IBA1 and iNOS in WT and KO mice. TBI‐mediated CD4 protein production was attenuated by naltrexone in WT mice, but not in KO mice.

**Conclusion:**

Naltrexone reduced TBI‐mediated neurodegeneration and inflammation in MOR WT and KO mice. The protective effect of naltrexone involves non‐MOR and MOR mechanisms.

## Graphical abstract

Naltrexone reduced glutamate or TBI‐mediated neurodegeneration and inflammation in MOR KO and WT mice.

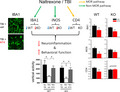

## 1 INTRODUCTION

Traumatic brain injury (TBI) is a global health problem and a major cause of long‐term disability and death among all trauma‐related injuries.^1^, ^2^ An estimated 5.3 million Americans live with a disability due to TBI (from the report to congress, traumatic brain in the United States, Centers for Disease Control and Prevention;www.cdc.gov/traumaticbraininjury/pubs/tbi_report_to_congress.html). The direct and indirect cost of TBI was around $48.3 billion annually in the United States^3^ and €33 billion in Europe for 2010.^4^, ^5^ Up to now, there is no effective pharmacological therapy for TBI in patients. It is critical to develop new treatments for TBI.

TBI induces primary injury directly from the traumatic impact,^6^ followed by a secondary injury from the response to the impact.^7^, ^8^ Inflammation is a major cause of secondary injury.^9^ TBI upregulates the expression of cytokines and chemokines, activates microglia, and migrates peripheral immune cells to the lesioned brain.^10^ In mice receiving moderate TBI, the levels of IL‐1β, tumor necrosis factor‐alpha, and IL‐6 peaked at 3‐9 hours post‐injury in the cortex.^11^ Similarly, the expression of IL‐6, IL‐8, IL‐10, and TNF‐alpha peaked in 2 days after moderate‐severe TBI in patients.^12^, ^13^ These inflammatory responses lead to apoptosis, gliosis, and neurodegeneration.^14^, ^15^, ^16^, ^17^ It is thus likely that the secondary injury of TBI can be modulated by anti‐inflammation‐based therapy.

Mu opioid receptor (MOR) antagonists have been used to treat drug dependence in patients. Selective MOR antagonists were found to possess neuroprotective or anti‐inflammatory activity. For example, naltrexone (brand names include ReVia and Vivitrol), a medication primarily used to manage alcohol or opioid dependence, reduced the expression of proapoptotic proteins BAD and BAX in the mouse brain.^18^ Naloxone attenuated lipopolysaccharide‐mediated nitric oxide production and TNF‐alpha expression in cortical neuron/glia co‐cultures^19^ and mitigated H_2_O_2_‐mediated neuronal loss in NSC34 cell culture.^7^ A meta‐analysis indicated that early treatment with naloxone reduced mortality and improved prognosis in patients with severe TBI.^20^ Interestingly, the protective action of MOR antagonists was also found in their (+) stereoisomers, which does not interact with MOR. Intranasal delivery of (+) naloxone reduced microglia activation and promoted behavioral recovery in stroke rats.^21^ (+) Naloxone was equally effective as (−) naloxone in inhibiting LPS‐mediated microglia activation in culture.^19^ These data suggest that naloxone may reduce neuroinflammation through non‐MOR mechanisms.

The purpose of this study was to characterize the neuroprotective action of naltrexone in a controlled cortical impact (CCI) model of TBI. MOR knockout (KO) and wild‐type (WT) mice were used to identify the specific action of the MORs. The KO mice had no detectable MOR.^22^, ^23^ The binding and function of delta or kappa opioid receptors in the KO mice were not affected.^23^ We demonstrated that naltrexone suppressed TBI‐mediated bradykinesia and altered microglia activation in both KO and WT mice. Naltrexone differentially inhibited TBI‐mediated CD4 expression in KO and WT. Our data suggest that naltrexone reduced inflammation and neurodegeneration through non‐MOR and MOR‐mediated mechanisms.

## 2 MATERIALS AND METHODS

### 2.1 Animals

MOR KO and WT mice were kindly provided by Dr. Horace H Loh.^23^ The animals were bred at the National Health Research Institutes (NHRI). The use of animals was approved by the Animal Research Committee of the NHRI (approved number: 109097A, 108146A). All animal experiments were carried out in accordance with the National Institutes of Health guide for the care and use of Laboratory Animals (NIH Publications No. 8023, revised 1978). All mice were kept in an animal room with a 12‐h light/dark cycle at a temperature of 25 ± 2°C and humidity of 55%. A standard diet and water were provided ad libitum.

### 2.2 Primary mouse cortical neurons and BV2 microglia co‐cultures

Primary cortical neurons (PCNs) and BV2 microglia co‐cultures were prepared, as we previously described.^24^ Cerebral cortical cells were obtained from E14‐15 fetuses of timed pregnant WT or KO mice. After removing the blood vessels and meninges, pooled cortices were trypsinized (0.05%; Invitrogen, Carlsbad, CA) for 20 min at room temperature. After rinsing with pre‐warmed Dulbecco's modified Eagle's medium (Invitrogen), cells were dissociated by trituration, counted, and plated into 96‐well (5.0 × 10^4^/well) cell culture plates precoated with poly‐D‐lysine (Sigma‐Aldrich, St. Louis, MO, USA). The culture plating medium consisted of neurobasal medium supplemented with 2% heat‐inactivated fetal bovine serum (FBS, Hyclone, Utah, USA), 0.5 mM L‐glutamine (Sigma‐Aldrich, St. Louis, MO, USA), 0.025 mM L‐glutamate (Sigma‐Aldrich, St. Louis, MO, USA), and 2% B27 (Invitrogen, Carlsbad, CA). Cultures were maintained at 37°C in a humidified atmosphere of 5% CO_2_ and 95% air. The cultures were fed by exchanging 50% of media with feeding media (Neurobasal Medium, Invitrogen) with 0.5 mM L‐glutamate and 2% B27 with an antioxidant supplement on days in vitro (DIV) 3 and 5. BV2 microglia were cultured separately, detached by 0.05% trypsin‐ethylene diamine tetraacetic acid (EDTA, Invitrogen, Carlsbad, CA), and centrifuged at 100 g for 5 min. BV2 cells were resuspended in the feeding media containing B27 supplement without antioxidants (‐AO, from Invitrogen). The density of surviving cells was counted using a trypan blue assay; cells were plated on the PCN‐plated wells at a concentration of 3.0x10^3^/well on DIV 7. The co‐cultures were fed with ‐AO media on DIVs 7 and 10. On DIV 10, cultures were treated glutamate with naltrexone or vehicle. At 48 hr after drug treatment, cells were fixed 4% paraformaldehyde (PFA, Sigma‐Aldrich, St. Louis, MO, USA) for 1 hr at room temperature.

### 2.3 Immunocytochemistry

Cultured cells were fixed with PFA for 1 hr, washed with PBS, and incubated with a mouse monoclonal antibody against MAP2 (1:500) or a rabbit polyclonal antibody against IBA1 (1:500) for 1 day at 4°C. The bound primary antibodies were later interacted with secondary antibodies (Alexa Fluor 488 goat anti‐mouse or Alexa Fluor 568 goat anti‐rabbit antibody, Invitrogen). Images were acquired by a DS‐Qi2 camera (Nikon, Melville, NY) attached to a NIKON ECLIPSE Ti2 (Nikon, Melville, NY). Data were analyzed using NIS Elements AR 5.11 Software (Nikon).

### 2.4 Controlled cortical impact (CCI) and naltrexone injection

Adult male MOR WT and KO mice are anesthetized with isoflurane and placed in a stereotaxic frame. A midline incision was made to expose the skull, and a 4 mm craniotomy was made centered at −2 mm posterior to bregma and 0.5 mm lateral to midline over the left hemisphere. Mice were subjected to CCI at a 1.0 mm impact depth and a nominal velocity of 5 m/s. The dwell time was 500 ms, and the tip size was 2 mm. A computer‐controlled pneumatically driven piston from the CCI impactor device (TBI‐0310 Impactor, Precision Systems and Instrumentation, Fairfax Station, VA) was used to impact the brain. After the impact, the head wound was sutured. Body temperature was maintained at 37°C using a temperature‐controlled incubator. Control animals received sham surgery, including craniotomy without cortical impact. Naltrexone (Sigma, Cat. No: N3136, 10 mg/kg/d) or vehicle was given subcutaneously from day 2 to day 5 after CCI.

### 2.5 Behavioral test

Locomotor activity was examined on day 5 after CCI. Mice were individually placed in 42 × 42 × 26 cm Plexiglas activity chambers containing horizontal and vertical infrared sensors (Accuscan, Columbus, OH) placed 2.5 cm apart. Two variables were measured: (i) horizontal activity (HACTV, the total number of beam interruptions that occurred in the horizontal sensors in one hour) and (ii) vertical activity (VACTV, the total number of beam interruptions that occurred in the vertical sensor in one hour).

### 2.6 Immunohistochemistry

Brains were removed and dissected, post‐fixed in 4% paraformaldehyde (PFA) for 48 hr, and transferred to 20% sucrose in 0.1 M phosphate buffer (PB) for at least 16 h. Serial sections of the entire brain were cut at 25 μm thickness on a freezing cryostat (Leica Model: CM 3050 S). After blocking with 4% bovine serum albumin (Sigma‐Aldrich, St. Louis, MO, USA) and 0.3% Triton X‐100 in 0.1 M PB, sections were incubated with antibodies against ionized calcium‐binding adaptor molecule (polyclonal, IBA1; 1:100; Abcam, Cambridge, MA, USA) and kept at 4°C overnight. Control sections were incubated without the primary antibody. Sections were washed three times with 0.1 M PB and incubated in Alexa Fluor 488 goat anti‐mouse IgG (1:500, Thermo Fisher Scientific, Waltham, MA, USA) for 60 min at room temperature. Sections were mounted on slides and cover‐slipped. Confocal analysis was performed using a Nikon D‐ECLIPSE 80i microscope (Nikon Instruments, Inc., Tokyo, Japan) and the EZ‐C1 3.90 software (Nikon, Tokyo, Japan). The optical density of IBA1 immunoreactivity was quantified in three consecutive brain sections with a visualized anterior commissure in each animal, as previously reported.^25^ Five photomicrographs were taken along the perilesioned region per brain slice.

### 2.7 Western blotting

The right and left cerebral cortices were collected. Tissue was homogenized in RIPA lysis buffer (Merck Millipore, MA, USA) and then was centrifuged at 13 200 rpm for 10 min at 4°C. The supernatant was collected. A bicinchoninic acid (BCA) protein assay was performed to determine protein concentrations. The samples were diluted with RIPA buffer according to the BCA protein assay. Gels were transferred to a PVDF membrane (PerkinElmer, Waltham, MA) after electrophoresis. The membranes were blocked in 5% milk at room temp for 1 hr. The blots were then probed with primary antibodies against ionized calcium‐binding adapter molecule 1 (polyclonal, IBA1, 1: 500, Wako, Osaka, Japan), inducible nitric oxide synthase (monoclonal, iNOS, 1:1000, BD, San Jose, CA), CD4 (polyclonal, 1:500, Proteintech, IL, USA), or actin (monoclonal, 1: 10,000, Novus, CO, USA) at 4°C for overnight. The membrane was then incubated with horseradish peroxidase (HRP)–conjugated secondary antibody (Jackson laboratory) at room temp for 1 hr, followed by washing with 0.1% Tween‐20 (in PBS) three times for 10 min each. The light emission signal of the target proteins on the PVDF membrane was generated by using a Western Lightning Plus‐ECL (PerkinElmer, MA, USA) and then detected by X‐ray film (Cat. No. GE28‐9068–39, GE, Boston, USA). The amount of IBA1 and iNOS was normalized with actin on the same membrane. Band intensity was quantified using ImageJ.

### 2.8 Quantitative reverse transcription polymerase chain reaction (qRT‐PCR)

Cerebral cortical tissues were collected for qRT‐PCR analysis. Total RNAs were isolated using TRIzol Reagent (Thermo Fisher, #15596‐018, MA, USA), and cDNAs were synthesized from 1 ug total RNA using RevertAid H Minus First Strand cDNA Synthesis Kit (Thermo Scientific, #K1631). MOR and GDNF mRNA expression was measured by using SYBR Green (Luminaris Color HiGreen Low ROX qPCR Master Mix; Thermo Scientific, MA, USA) or TaqMan Fast (Life Technologies). Quantitative real‐time PCR (qRT‐PCR) was carried out using TaqMan Fast Advanced Master Mix (Life Technologies, #4444557) and QuantStudio^TM^ 3 Real‐Time PCR System (Thermo Scientific). The expression of target genes was normalized to the reference genes (beta‐actin and GAPDH average) with a modified delta‐delta‐Ct algorithm. All experiments were duplicated. The primers for GDNF, MOR, beta‐actin, and GAPDH are listed in Table 1.

**TABLE 1:** Oligonucleotide primers used for qRT‐PCR

Gene	SYBR Green		TaqMan
	Forward	Reverse	
GDNF	TAAGATGAAGTTATGGGATGTCG	CTTCGAGAAGCCTCTTACCG	universal probe Library #, Roche 112
MOR	ACTGGGAGAACCTGCTCAAA	GGGGTCCAGCAGACAATAAA	
β‐Actin			Mm02619580_g1
GAPDH			Mm99999915_g1

### 2.9 Statistical analysis

Data were presented as mean ±SEM. The normality of variables was examined by the Shapiro‐Wilk test. Data that did not exhibit a normal distribution were analyzed via a non‐parametric equivalent. One‐ or two‐way ANOVA and post hoc Newman‐Keuls tests (NK test) were used for statistical comparisons, with a significance level of *P *< 0.05.

## 3 RESULTS

### 3.1 Naltrexone induced neuroprotection in neuron/microglia co‐culture

**FIGURE 1:**
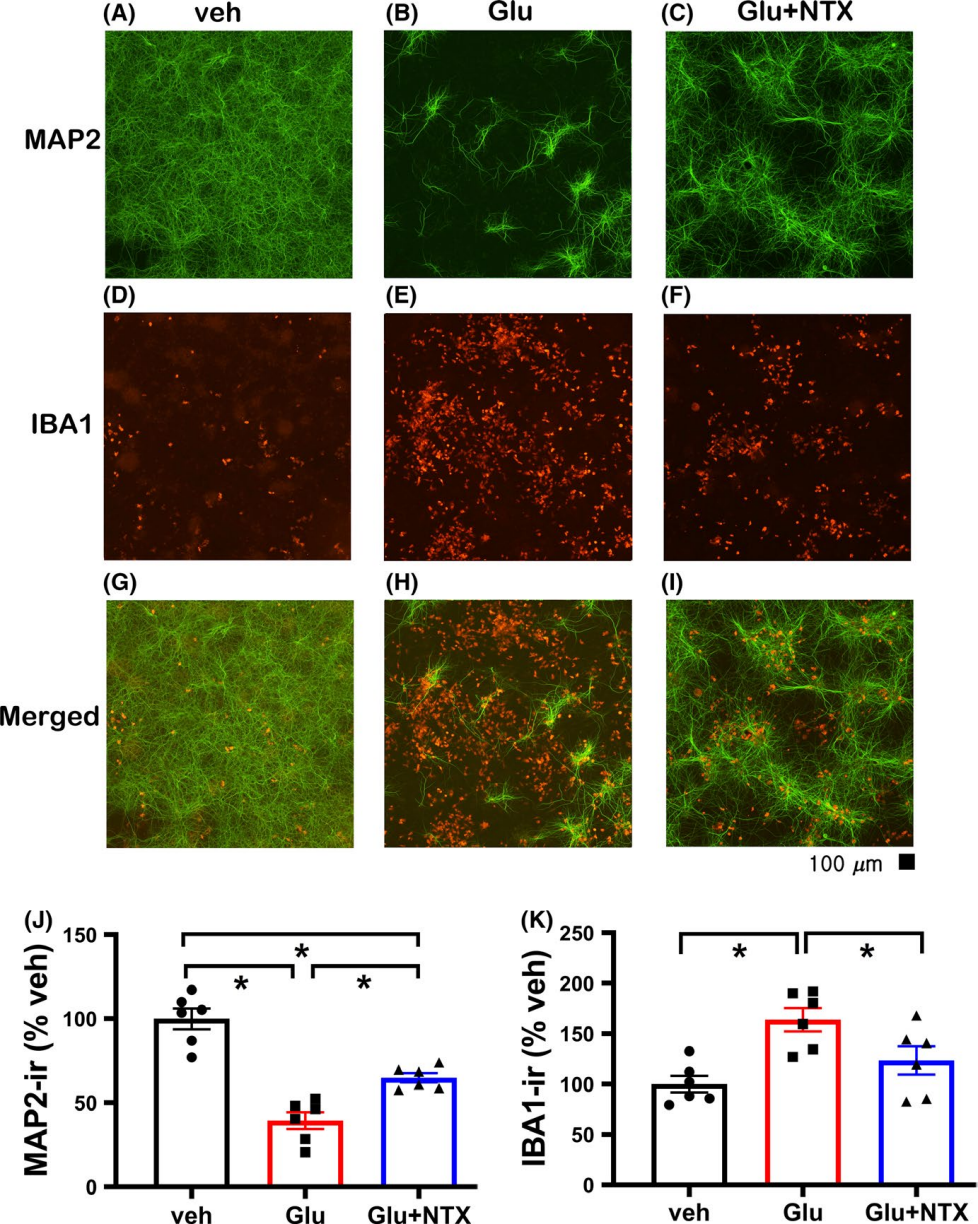
Naltrexone antagonized glutamate‐mediated neuronal loss and microglia activation in neuronal‐microglia co‐culture. Primary cortical neuronal cells from MOR WT embryos were co‐cultured with BV2 microglia. Glutamate (Glu) treatment reduced MAP2‐ir (B vs A), while increased IBA1‐ir (E vs D). Both responses were antagonized by naltrexone (C, F, J, and K). (G: merged A and D, H: merged B and E, and I: merged C and F.) Scale bar = 100 μm. * *P* < 0.05, one‐way ANOVA +post hoc NK test. Data are represented as mean ± SEM. NTX: naltrexone

Primary cortical neurons (PCNs) from WT mouse embryos were co‐cultured with BV2 microglial as previously described.^24^ Glutamate (Glu)‐mediated neuronal loss was examined by MAP‐2 immunostaining. Glu (15 µM, n = 6) significantly reduced MAP2‐ir (Figure 1B vs 1A; Figure 1J, *P*<0.001); this response was antagonized by naltrexone (10 µM; Figure 1C and J, *P*<0.001, F_2, 15_ = 39.022, one‐way ANOVA+NK test). Glu enhanced IBA1‐ir (Figure 1E vs 1D; Figure 1K, *P* = 0.004), which was also antagonized by naltrexone (Figure 1F and K, *P* = 0.025, F_2, 15_ = 7.946, one‐way ANOVA+NK test).

**FIGURE 2:**
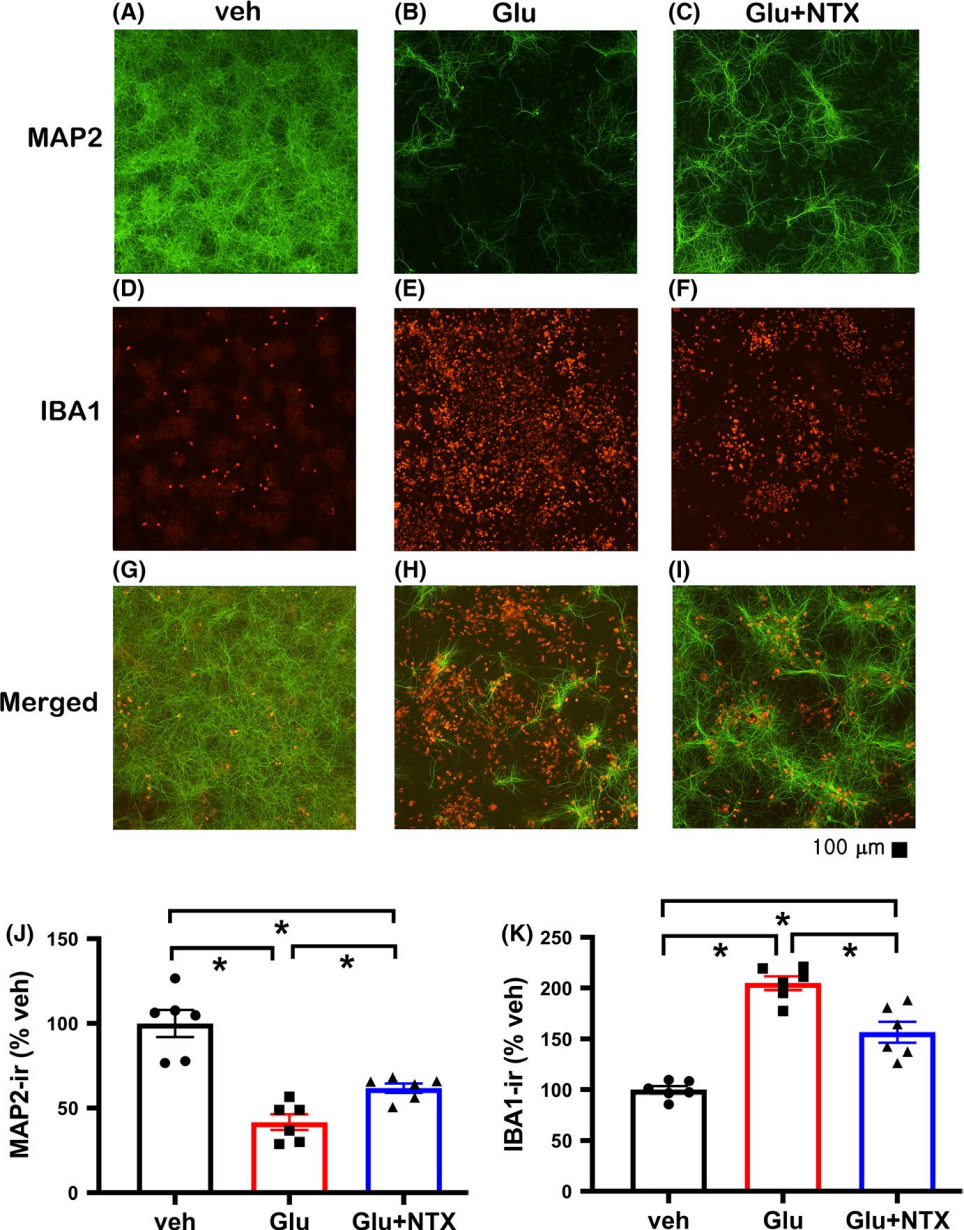
Naltrexone antagonized glutamate‐mediated neuronal loss and inflammation in the co‐culture of PCNs from MOR KO and BV2 microglia. Treatment with glutamate (Glu) reduced MAP2‐ir (B vs A), while increased IBA1‐ir (E vs D). Both responses were antagonized by naltrexone (C and F). One‐way ANOVA indicated that naltrexone significantly antagonized the Glu‐mediated reduction of MAP2‐ir (J) and increase in IBA1‐ir (K). (G: merged A and D, H: merged B and E, and I: merged C and F.) Scale bar = 100 μm. NTX: naltrexone

We next examined whether knocking out MOR receptors altered the protective action of naltrexone. PCNs from MOR KO embryos were co‐cultured with BV2 microglia. Glu (15 µM, n = 6) significantly reduced MAP2‐ir (Figure 2B, *P* = <0.001) and enhanced IBA1‐ir (Figure 2E, *P* = <0.001); both responses were significantly antagonized by naltrexone (MAP2: Figure 2C and J, *P* = <0.001, F_2,15_ = 28.578; IBA1: Figure 2F and K, *P* = <0.001, F_2,15_ = 50.818, one‐way ANOVA +NK test).

### 3.2 Naltrexone improved locomotor activity in KO and WT mice

**FIGURE 3:**
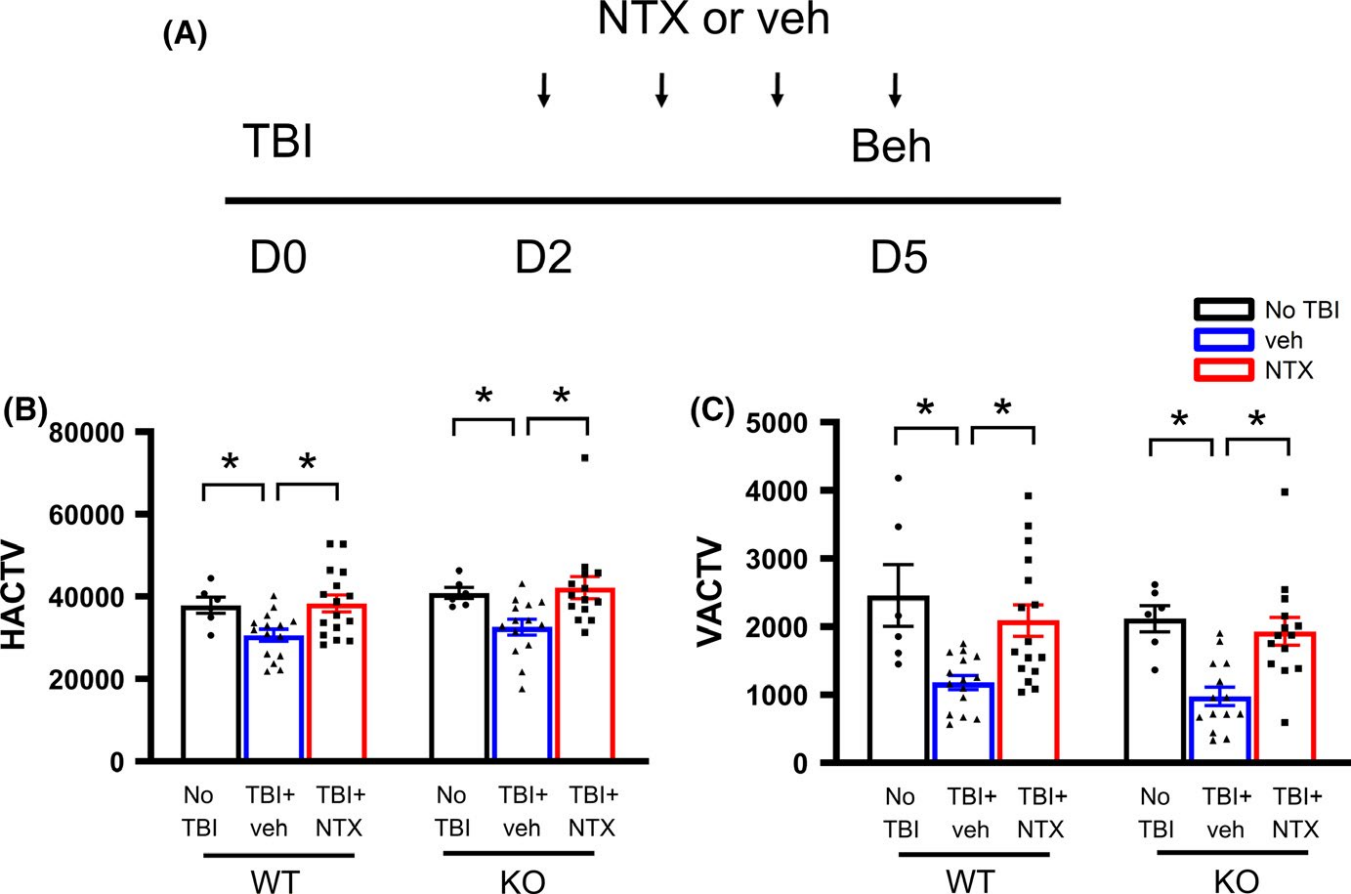
Naltrexone improved locomotor behavioral function in MOR KO and WT mice after TBI. Mice received naltrexone (10 mg/kg/d, s.c.) on days 2–5 after TBI. (A) Locomotor activity was examined on D5. TBI significantly reduced (B) horizontal activity (HACTV) and (C) vertical activity (VACTV) in WT and KO mice. Naltrexone significantly improved HACTV and VACTV. * *P* < 0.05, two‐way ANOVA +post hoc NK test. Data are represented as mean +/‐ SEM

A total of 31 WT and 28 MOR KO mice received CCI or sham surgery on day 0 and were treated with vehicle (WT: n = 15; MOR KO n = 14) or naltrexone (WT: n = 16; MOR KO n = 14. 10 mg/kg/d, s.c.) from days 2 to 5. Locomotor activity was examined on day 5. TBI significantly reduced horizontal activity (HACTV) and vertical activity (VACTV) in the WT and KO mice. (Figure 3, Table 2). No difference was found between WT and KO (Table 2, two‐way ANOVA). Treatment with naltrexone normalized HACTV and VACTV in the lesioned WT and KO mice (Figure 3, Table 2).

**TABLE 2:** Significant differences in locomotor behaviors among all groups on D5 post‐TBI

	WT		KO		no TBI	TBI+veh	TBI+NTX
	TBI vs no TBI	TBI vs TBI +NTX	TBI+veh vs no TBI	TBI +veh vsTBI+NTX	WT vs KO	WT vs KO	WT vs KO
HACTV	*P* = 0.047	*P* = 0.014	*P* = 0.027	*P* = 0.003	*P* = 0.495	*P* = 0.473	*P* = 0.166
VACTV	*P* = 0.001	*P*<0.001	*P* = 0.005	*P*<0.001	*P* = 0.419	*P* = 0.449	*P* = 0.544

Abbreviations: HACTV: horizontal activity; VACTV: vertical activity and NTX: naltrexone.

*P*‐value was determined by a two‐way ANOVA +NK test.

### 3.3 Naltrexone reduced TBI‐mediated microglial activation in WT and KO mice

**FIGURE 4:**
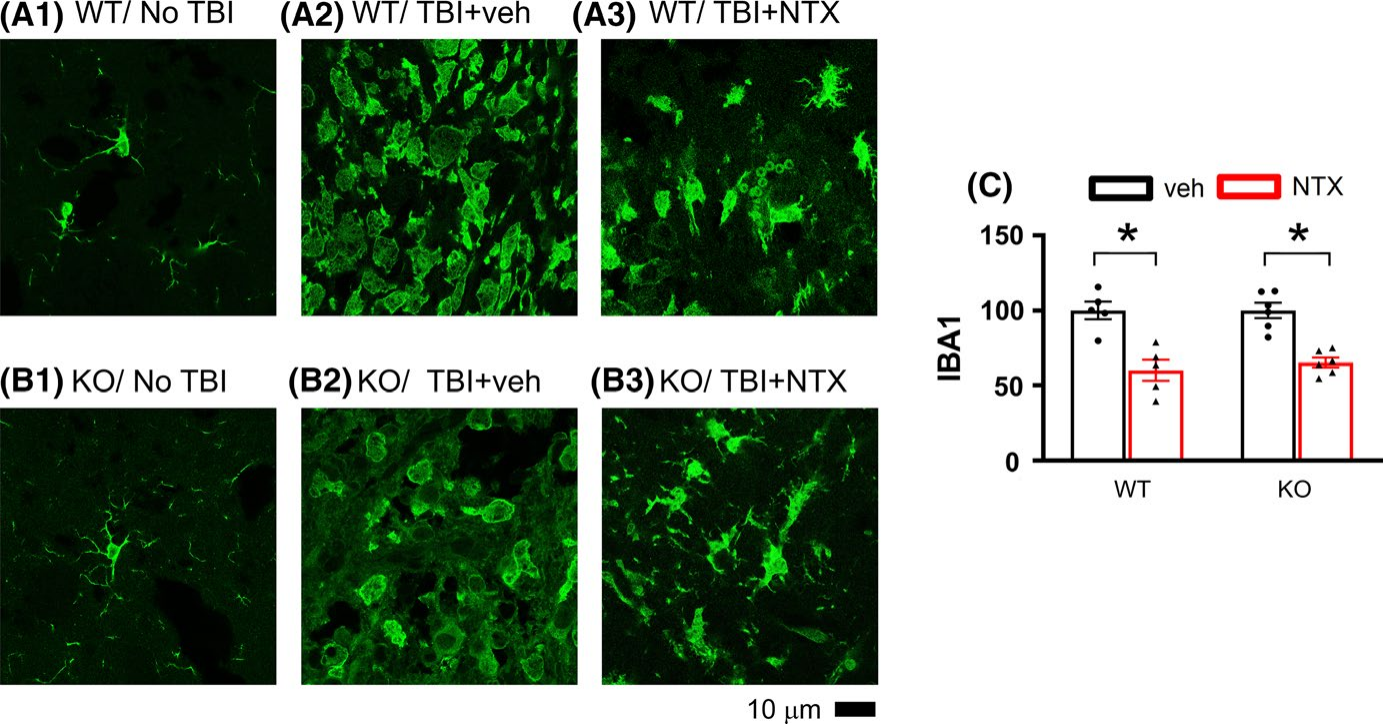
Naltrexone reduced TBI‐mediated microglial activation in the lesioned cortex. (A) WT and (B) KO mice received TBI and naltrexone (10 mg/kg/d, s.c. from days 2 to 5) or vehicle. Animals were sacrificed on day 5. Enhanced IBA1 immunoreactivity with deramified morphology was found in the lesioned cortex in WT (A2 vs A1) and KO (B2 vs B1) after injury. Treatment with naltrexone reduced IBA immunoreactivity (WT: A3 vs A2; KO B3 vs B2). (C) Naltrexone significantly mitigated IBA1‐ir in the TBI‐WT and TBI‐KO mice. Scale bar = 10 µm, *P<0.001, two‐way ANOVA +post hoc NK test. NTX: naltrexone

A total of 22 mice were used for IBA1 immunostaining. Of these, 11 mice (5 WT +6 KO) received TBI, followed by vehicle injection. The other 11 mice (5 WT +6 KO) received naltrexone after TBI. Brain tissues were collected on day 5. Enhanced IBA1‐ir and deramified microglial morphology were found in the perilesioned area of WT (Figure 4 A2) and KO (Figure 4 B2) mice receiving vehicle. Resting microglia exhibiting ramified morphology were found in the contralateral (non‐lesioned side) cortex (Figure 4 A1 and B1). TBI‐enhanced IBA1 immunoreactivity and microglia activation were antagonized by naltrexone in the WT and KO mice (WT: Figure 4 A3 and KO: Figure 4 B3). The optical density of IBA1 immunofluorescence in the perilesion zone was quantified and averaged from 5 images taken from the brain slices with visible anterior commissures. IBA1‐ir was significantly enhanced in the lesioned brain. No difference was found between the WT and KO (*P* = 0.219). Naltrexone significantly reduced IBA1 optical density in WT (*P*<0.001, two‐way ANOVA+NK test) and KO mice (Figure 4C, *P*<0.001).

### 3.4 TBI increased IBA1, iNOS, and CD4 protein levels in WTs and KOs

**FIGURE 5:**
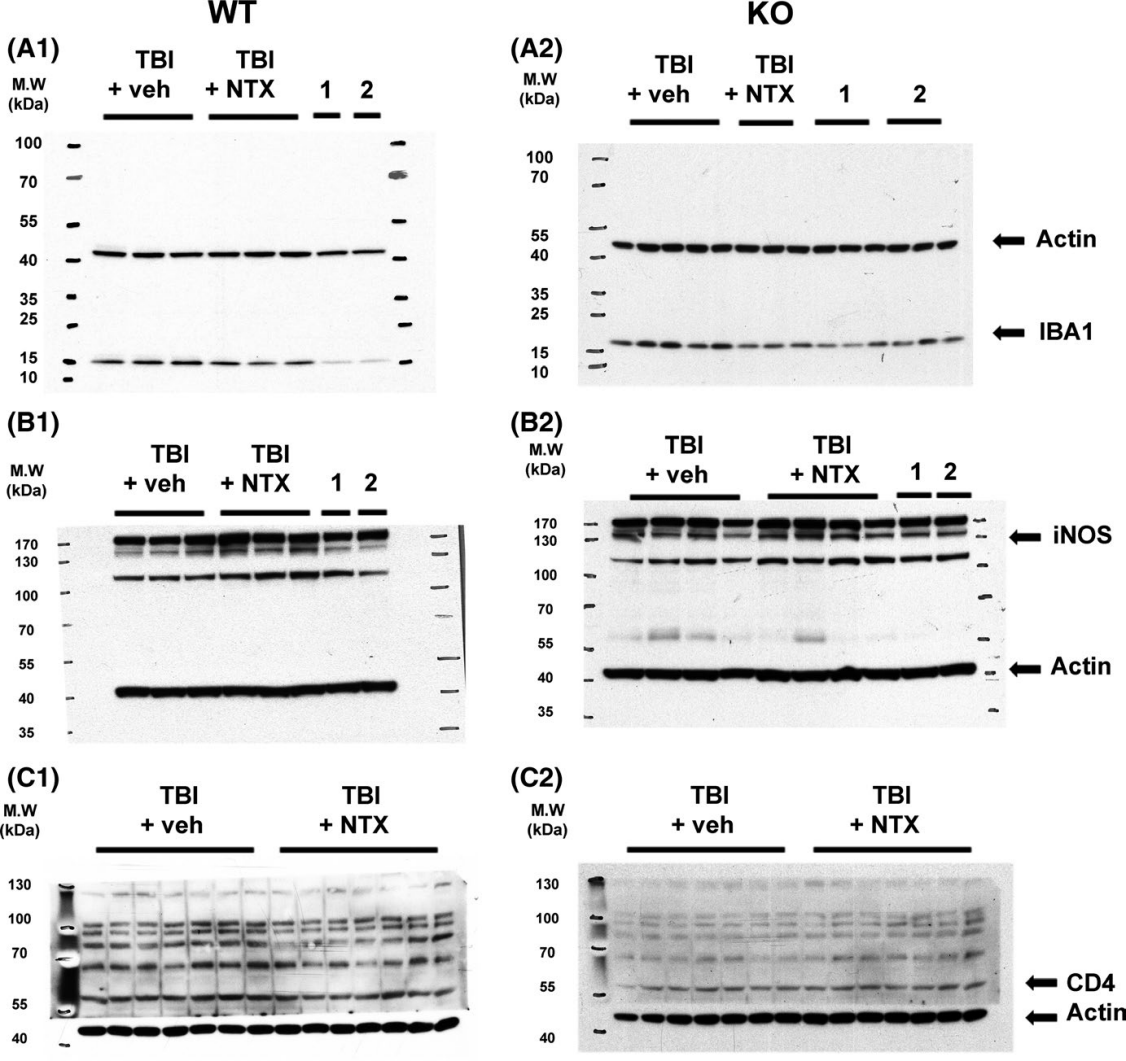
Representing Western blots of IBA1, iNOS, and CD4 proteins in the lesioned (TBI) and non‐lesioned (1: no TBI +NTX; 2: no TBI +veh) cortices. Naltrexone (NTX) reduced TBI‐mediated IBA1, iNOS, and CD4 in the WT (A1, B1, C1) mice. Naltrexone also antagonized TBI‐mediated expression of IBA1 and iNOS in the KO mice (A2 and B2), but not CD4 (C)

**FIGURE 6:**
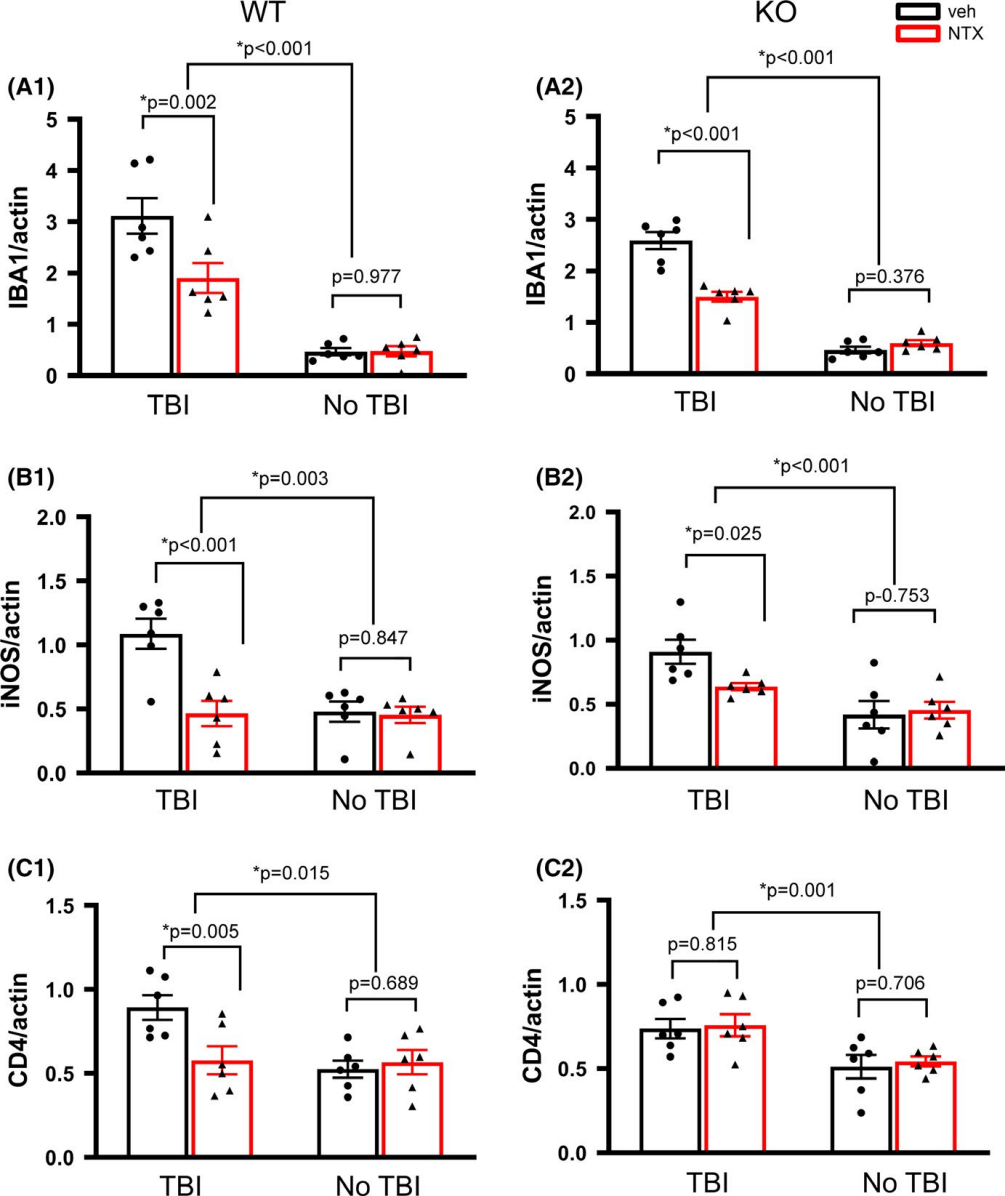
The expression of IBA1, iNOS, and CD4 proteins in the lesioned (TBI) and non‐lesioned (no TBI) cortices of WT and KO mice. The expression of (A) IBA1, (B) iNOS, and (C) CD4 was examined by Western analysis and normalized to actin. TBI significantly increased IBA1 (WT: A1, KO: A2), iNOS (WT: B1, KO: B2), and CD4 (WT: C1, KO: C2). Naltrexone (NTX) significantly reduced TBI‐induced IBA1 and iNOS in the WT (A1, B1) and KO (A2, B2) mice. Naltrexone significantly antagonized TBI‐mediated CD4 expression in the WT mice (C1), but not KO mice (C2). N = 6 in each group. Two‐way ANOVA +post hoc NK test

The lesioned and non‐lesioned side cortices were collected from 12 WT and 12 KO mice on day 5 post‐TBI for Western analysis (Figure 5). TBI significantly increased IBA1 (Figure 6 A1, *P*<0.001, WT; Figure 6 A2, *P*<0.001, KO, two‐way ANOVA +NK test), iNOS (Figure 6 B1, *P* = 0.003, WT; Figure 6 B2, *P*<0.001, KO), and CD4 protein levels (Figure 6 C1, *P* = 0.015, WT; Figure 6 C2, *P* = 0.001, KO) in the lesioned brains. Naltrexone significantly mitigated TBI‐induced IBA1 (WT: Figure 6 A1, *P* = 0.002; KO: Figure 6 A2, *P*<0.001) and iNOS (WT: Figure 6 B1, *P*<0.001; KO: Figure 6 B2 *P* = 0.025). No difference was found between KO and WT mice (IBA1: *P* = 0.258, Figure 6 A1 vs A2; iNOS, *P* = 0.197, Figure 6 B1 vs B2, two‐way ANOVA+NK test). Naltrexone selectively antagonized TBI‐mediated CD4 expression in the WTs (*P* = 0.005, Figure 6 C1), but not in the KOs (*P* = 0.815, Figure 6 C2).

### 3.5 Naltrexone did not alter the expression of MOR and GDNF

**FIGURE 7:**
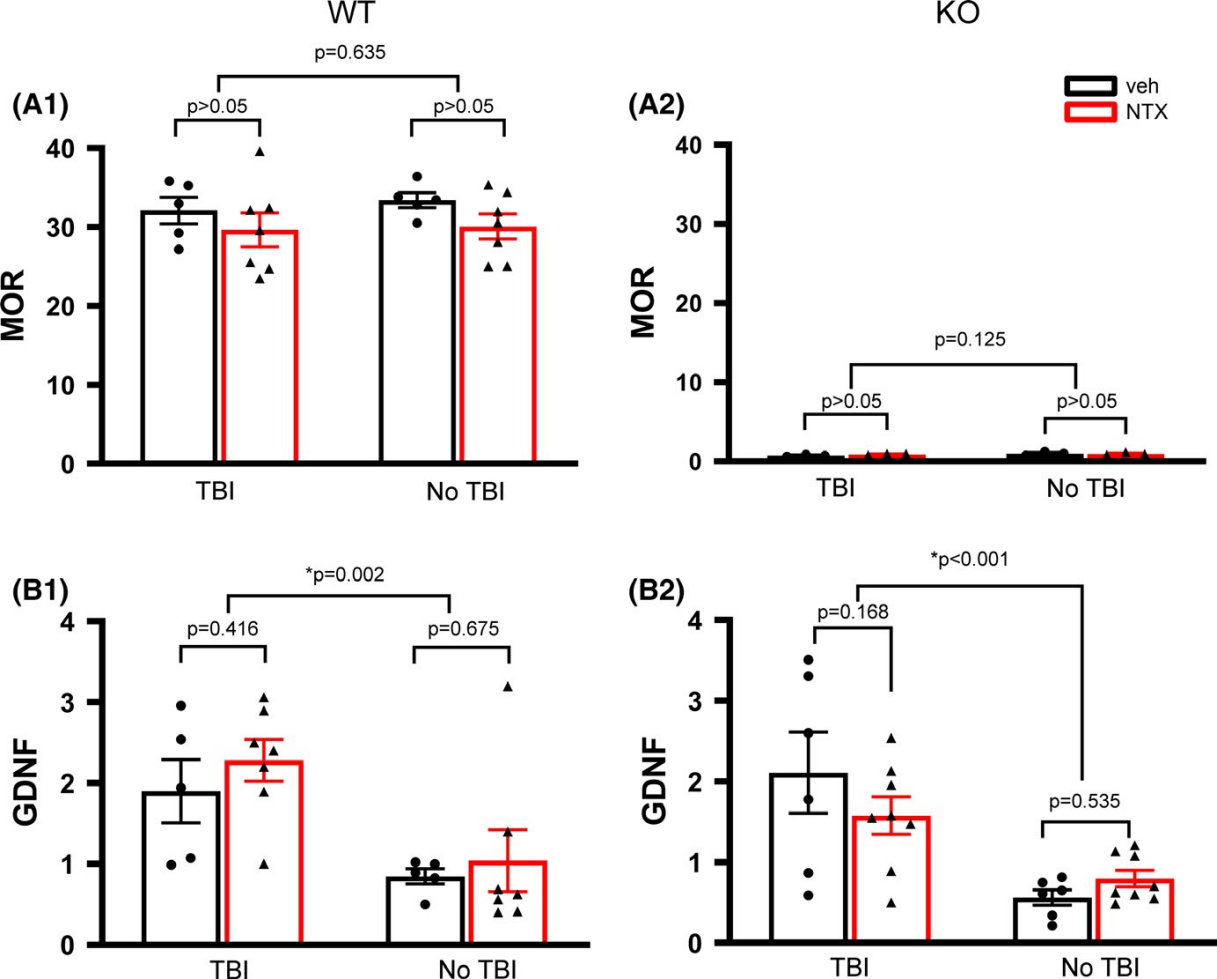
Naltrexone did not alter expression of MOR or GDNF in WT or KO mice. The expression of (A1 for WT; A2 for KO) MOR and (B) GDNF in lesioned (TBI) and non‐lesioned side (no TBI) cerebral cortex was examined by qRT‐PCR. The expression of target genes was normalized to the reference genes (beta‐actin and GAPDH average) with a modified delta‐delta‐Ct algorithm. TBI significantly increased the GDNF expression (TBI vs no TBI) in WT (B1) and KO (B2) mice. Treatment with naltrexone did not alter the expression of (A) MOR or (B) GDNF in WT or KO mice

Brain tissues from 12 WT and 14 KO mice were collected on day 5 for qRT‐PCR analysis. As expected, no detectable MOR mRNA was found in the KO mice (*P*<0.001, WT vs KO). TBI or naltrexone treatment did not alter the MOR expression in WT mice (Figure 7 A1. TBI vs non‐TBI: *P* = 0.635; naltrexone vs vehicle: *P*>0.05, two‐way ANOVA).

TBI significantly increased GDNF expression in WT (Figure 7 B1, *P* = 0.002) and KO (Figure 7 B2, *P*<0.001). Naltrexone did not alter expression of GDNF (WTs: Figure 7 B1, *P* = 0.416; KOs: Figure 7 B2: *P* = 0.168).

## 4 DISCUSSION

Naltrexone‐induced neuroprotection was examined in neuron/microglia co‐culture and a mouse model of TBI. We demonstrated that naltrexone significantly reduced glutamate‐mediated neuronal loss and microglia activation in the co‐culture. In both MOR WT and KO mice, early post‐treatment of naltrexone improved locomotor activity, while reduced microglia activation and iNOS expression after TBI. Naltrexone selectively inhibited TBI‐mediated CD4 expression in WT mice. The main finding in this study is that naltrexone induced protection in the TBI brain through anti‐inflammation.

Several studies have supported that MOR agonists modulate neuroinflammation after brain injury.^26^ For example, morphine increased NF‐κB levels in LPS‐activated microglia. This response is selective to MOR as NF‐κB was also activated by MOR agonist DAMGO, but not with delta or kappa opioid agonists DPDPE or U69593.^27^ Furthermore, transfection with siRNAs that target MOR mRNA antagonized NF‐κB activation.^27^ These data suggest that activation of MOR enhances neuroinflammation and degeneration.

In this study, we examined the interaction of MOR antagonist naltrexone after injury in cultured cells and an animal model of TBI. We demonstrated that naltrexone significantly mitigated microglia activation in neuron/microglia co‐culture and reduced IBA1 and iNOS expression, as well as behavior deficits, in the TBI mice. Similar protective responses have been reported. Naltrexone attenuated the expression of BAD and BAX in mouse brain.^18^ These data suggest that naltrexone is neuroprotective against TBI‐mediated neuroinflammation and neurodegeneration.

TBI can lead to chronic neurodegeneration and long‐term neurological deficits.^28^, ^29^, ^30^ For example, neurological severity scores, rotarod latency, impairments of cognitive function in Y or Water maze,^31^, ^32^ foot faults in motor function test,^33^ and forelimb asymmetry^32^ were increased and lasted up to 28‐35 days after CCI in mice. A few compounds have been reported to mitigate these secondary injuries.^31^ Similar responses have also been reported in other brain injuries.^21^ Neuronal loss and neuroinflammation were found at weeks after stroke in rats.^21^ (+)‐Naloxone antagonized the delayed microglia/macrophage activation and behavioral deficits in chronic stroke rats. ^21^ In this study, we demonstrated that naltrexone reduced inflammation and improved locomotor behavior at 5 days after TBI. Naltrexone may also reduce the delayed neurodegeneration in the TBI brain, which warrants further investigation.

The role of endogenous opioids in TBI was characterized in the MOR knockout mice. These animals did not express MORs, as confirmed by qRT‐PCR (Figure 7); the Kd and Bmax for kappa or delta opioid receptors were not affected.^23^ We found that TBI induced a similar upregulation of IBA1 and iNOS in the lesioned cortex, as well as behavioral deficits, in the MOR WT and KO mice receiving vehicle. Similar neurodegenerative/inflammatory change was found in WT and KO neuron/microglia co‐cultures after challenging with glutamate. These data suggest that knocking out MOR did not alter endogenous protection against TBI‐mediated neurodegeneration.

We found that naltrexone equally normalized behavioral deficits, microglia activation, and iNOS expression in WT and KO mice after TBI. Naltrexone also antagonized glutamate‐mediated MAP2 and IBA1 expression in WT and KO neuron/microglia cultures. These data suggest that MOR is not essential for naltrexone‐mediated protection, as no difference was found between WTs and KOs. The non‐opioid protective reaction of naltrexone or its analogs has been reported by other laboratories. (+) Naltrexone, which did not interact with MOR, reduced TLR2‐ and TLR4‐mediated nitric oxide release from BV2 microglia.^34^ Intranasal delivery of (+) naloxone reduced microglia activation and promoted behavioral recovery in stroke rats through a non‐MOR mechanism.^21^ These data support the non‐MOR action of naltrexone after brain injury.

Previous studies have indicated that MOR regulated lymphocyte activity in brain.^35^ Similar to previous reports,^36^ we found that TBI significantly increased the expression of lymphocyte marker CD4 in the lesioned brain. Naltrexone antagonized the upregulation of CD4 in WT, but not in the KO mice. This differential response of naltrexone in WT and KO suggests that MOR is involved in the migration of CD4+ lymphocytes to the TBI. The interaction of naltrexone and peripheral lymphocytes in TBI warrants further characterization.

GDNF is a neurotrophic factor for TBI and stroke. Ischemic brain injury upregulated the expression of GDNF^37^ and its receptor GFR‐alpha‐1.^38^ Administration of GDNF protein^39^ or upregulation of GDNF expression^40^ reduced brain infarction and neurological deficits in stroke rats. A few studies have suggested that opioids induce protection through GDNF. Delta opioid peptide [D‐ala2,D‐leu5] enkephalin (DADLE) increased GDNF expression and protected against cell death in stroke brain.^41^ We found that TBI increased the expression of GDNF in the lesioned brain. Administration of naltrexone or knocking out the MOR did not alter the expression of GDNF, suggesting that naltrexone did not induce protection through GDNF in the TBI brain.

We found that naltrexone, a drug that is commonly used for the treatment of alcohol and opioid abuse, has a protective and anti‐inflammatory effect in TBI mice. Our study is supported by a clinical report that a severe TBI patient developed functional improvement after naltrexone therapy.^42^ As a high incidence of TBI is associated with substance abuse and drug abuse exacerbates TBI's degenerative effects,^43^ naltrexone may be useful for the treatment of drug addiction and the comorbidity of TBI in drug abusers.

## 5 CONCLUSION

Our data support the notion that naltrexone reduced TBI‐mediated neurodegeneration and inflammation, likely through non‐MOR and MOR mechanisms.

## CONFLICT OF INTEREST

There are no competing interests.

## Data Availability

The data that support the findings of this study are available from the corresponding author upon reasonable request.
